# The Sins of Teetotalers

**Published:** 1898-07-30

**Authors:** 


### The Sins of Teetotalers.

The Midland Branch of the British Medical Association has the advantage of possessing a President, in the person of Dr. Carline, who, in addition to the acquirements and the reputation which have conducted him to that distinguished position, has been endowed by Nature with the inestimable gift of common sense, and with the courage necessary to make public the conclusions to which it leads. We have taken, as the title of this article, that of his Presidental address; and we desire to congratulate him, and still more those who had the pleasure of hearing him, upon the robustness of its character and upon the truth of its allegations. He pointed out, not for the first time, but none the less effectively, that the propaganda of teetotalism is purely destructive; and that the active members of the sect, or body, or whatever they call themselves, have never made the smallest attempt to provide a substitute for the alcohol which they condemn. In Lincoln, as Dr. Carline, speaking from complete local knowledge, points out, all endeavours to improve the water supply of the place have been opposed by teetotalers who have occupied official positions in relation to the subject, and have been neglected or ignored by those who were only private individuals. It has long been one of the commonplaces of sanitation that cholera, typhoid, and, probably, other diseases, find in polluted water their most ordinary channel of conveyance; and yet who ever heard of a teetotaler who, at the same time, was a convinced and earnest advocate for the protection and the improvement of the sources of supply? Such enterprises as those of the Salutaris Company, or of the Puralis Company, have never been actively encouraged by teetotalers as such; and the general insecurity of water drinking has passed into a proverb which they have done absolutely nothing to disprove, even in this paradise of faddists, the United Kingdom of Great Britain and Ireland. Abroad matters are still worse. We were once present when a very distinguished public man, on the eve of a journey to Italy, asked the late Sir Richard Quain how best to preserve his health during his absence. "Oh," was the reply, "there is not much to fear as long as you do not drink any water." The intending traveller was a somewhat hard man, not easily moved to any display of feeling, but the advice and the contained imputation were too much for him. "Quain," he said, speaking with a manifest effort and with a broken voice, "you have known me very intimately for twenty years. Did you ever see me touch it?" And, if not water, what then? The majority of the so-called "temperance drinks" are commonly supposed, rightly or wrongly, to contain, of course, for the purposes of preservation only, at least as much alcohol as those which they are designed to replace; and the abominations of the cottage teapot are sufficiently known to every practitioner whose lot it has been to attend to the out-patients of any great hospital. We entirely agree with Dr. Carline in his opinion that it should be the first duty of the preachers of abstinence to recognise the fact that a certain amount of fluid is a necessary of life, and to point out in what forms and in what quantities the fluid should be supplied. A policy that is merely destructive must of necessity be barren; and the congregations preached to by teetotal lecturers and advocates have a perfect right to inquire from such self-appointed guides: If not alcohol, what then? Sir James Paget, with a perfection of common sense which at least bordered upon genius, pointed out many years ago that in this country neither teetotalers or drunkards have ever formed more than a small section of the population. The enormous majority of Britons and of Irishmen are the descendants of many generations of moderate drinkers; and Britons and Irishmen are what they are, with their achievements in the past recorded in undying literature, and with the events of to-day showing that they have in no way fallen from the standards of former generations of their race. In some districts they have produced what is probably the most wholesome of beverages, cider; in others the national beer has been in greater favour; in all, as a rule, they have constantly consumed diluted alcohol in some of its infinitely varied forms. It may well be maintained that such experience as this, extending over many centuries and effecting countless multitudes of men and women, is of greater value than the results obtained by placing bits of tissue under the microscope of the pathologist.

